# Geographic clustering of testicular cancer incidence in the northern part of The Netherlands

**DOI:** 10.1038/sj.bjc.6690839

**Published:** 1999-12

**Authors:** D J A Sonneveld, M Schaapveld, D Th Sleijfer, G J Te Meerman, W T A van der Graaf, R H Sijmons, H Schraffordt Koops, H J Hoekstra

**Affiliations:** Departments of Surgical OncologyMedical Oncology Medical Genetics, Groningen University Hospital, PO Box 30.001, Groningen, RB, 9700, The Netherlands; Comprehensive Cancer Centre North-Netherlands, Groningen, The Netherlands

**Keywords:** testicular cancer incidence, geographic variation, urbanization, genetic susceptibility

## Abstract

Geographic variations in testicular cancer incidence may be caused by differences in environmental factors, genetic factors, or both. In the present study, geographic patterns of age-adjusted testicular cancer incidence rates (IRs) in 12 provinces in The Netherlands in the period 1989–1995 were analysed. In addition, the age-adjusted IR of testicular cancer by degree of urbanization was evaluated. Cancer incidence data were obtained from the Netherlands Cancer Registry. The overall annual age-adjusted IR of testicular cancer in The Netherlands in the period 1989–1995 was 4.4 per 100 000 men. The province Groningen in the north of the country showed the highest annual IR with 5.8 per 100 000 men, which was higher (*P* < 0.05) than the overall IR in The Netherlands (incidence rate ratio (IRR) 1.3, 95% confidence interval (CI) 1.1–1.6). The highest IR in Groningen was seen for both seminomas and non-seminomas. In addition, Groningen showed the highest age-specific IRs in all relevant younger age groups (15–29, 30–44 and 45–59 years), illustrating the consistency of data. The province Friesland, also situated in the northern part of the country, showed the second highest IR of testicular cancer with 5.3 cases per 100 000 men per year (IRR 1.2, 95% Cl 1.0–1.5, not significant). This mainly resulted from the high IR of seminoma in Friesland. Analysis of age-adjusted IRs of testicular cancer by degree of urbanization in The Netherlands showed no urban–rural differences at analysis of all histological types combined, or at separate analyses of seminomas and non-seminomas. Geographic clustering of testicular cancer seems to be present in the rural north of The Netherlands with some stable founder populations, which are likely to share a relatively high frequency of genes from common ancestors including genes possibly related to testicular cancer. Although this finding does not exclude the involvement of shared environmental factors in the aetiology of testicular cancer, it may also lend support to a genetic susceptibility to testicular cancer development. Testicular cancer cases in stable founder populations seem particularly suitable for searching for testicular cancer susceptibility genes because such genes are likely to be more frequent among affected men in such populations. © 1999 Cancer Research Campaign


*British Journal of Cancer* (1999) **81**(7), 1262–1267
© 1999 Cancer Research Campaign
Article no. bjoc.1999.0839

### Geographic clustering of testicular cancer incidence in the northern part of The Netherlands

**DJA Sonneveld[1], M Schaapveld[4], DTh Sleijfer[2], GJ Te Meerman[3], WTA van der Graaf[2], RH Sijmons[3], H Schraffordt Koops[1] and HJ Hoekstra[1]**

Departments of [1]Surgical Oncology, [2]Medical Oncology and [3]Medical Genetics, Groningen University Hospital, PO Box 30.001, 9700 RB Groningen, The Netherlands; [4]Comprehensive Cancer Centre North-Netherlands, Groningen, The Netherlands

**Summary** Geographic variations in testicular cancer incidence may be caused by differences in environmental factors, genetic factors, or both. In the present study, geographic patterns of age-adjusted testicular cancer incidence rates (IRs) in 12 provinces in The Netherlands in the period 1989–1995 were analysed. In addition, the age-adjusted IR of testicular cancer by degree of urbanization was evaluated. Cancer incidence data were obtained from the Netherlands Cancer Registry. The overall annual age-adjusted IR of testicular cancer in The Netherlands in the period 1989–1995 was 4.4 per 100 000 men. The province Groningen in the north of the country showed the highest annual IR with 5.8 per 100 000 men, which was higher ($P < 0.05$) than the overall IR in The Netherlands (incidence rate ratio (IRR) 1.3, 95% confidence interval (CI) 1.1–1.6). The highest IR in Groningen was seen for both seminomas and non-seminomas. In addition, Groningen showed the highest age-specific IRs in all relevant younger age groups (15–29, 30–44 and 45–59 years), illustrating the consistency of data. The province Friesland, also situated in the northern part of the country, showed the second highest IR of testicular cancer with 5.3 cases per 100 000 men per year (IRR 1.2, 95% CI 1.0–1.5, not significant). This mainly resulted from the high IR of seminoma in Friesland. Analysis of age-adjusted IRs of testicular cancer by degree of urbanization in The Netherlands showed no urban–rural differences at analysis of all histological types combined, or at separate analyses of seminomas and non-seminomas. Geographic clustering of testicular cancer seems to be present in the rural north of The Netherlands with some stable founder populations, which are likely to share a relatively high frequency of genes from common ancestors including genes possibly related to testicular cancer. Although this finding does not exclude the involvement of shared environmental factors in the aetiology of testicular cancer, it may also lend support to a genetic susceptibility to testicular cancer development. Testicular cancer cases in stable founder populations seem particularly suitable for searching for testicular cancer susceptibility genes because such genes are likely to be more frequent among affected men in such populations. © 1999 Cancer Research Campaign

**Keywords**: testicular cancer incidence; geographic variation; urbanization; genetic susceptibility

Geographic variations in cancer incidence rates (IRs) are not uncommon and may be caused by differences in environmental and/or genetic predisposing factors. Geographic clustering has been reported for numerous cancers including testicular cancer. The information available mainly showed geographic variations in nationwide IRs of testicular cancer between countries. The incidence is highest in Scandinavian countries (in particular in Denmark and with exception of Finland), Western European countries and New Zealand, intermediate in the US, and lowest in Asian countries (China, Hong Kong, India, Japan, Singapore) and African countries (Kenya, Nigeria) (Kolonel et al, 1982; Wilkinson et al, 1992; Adami et al, 1994; Buetow, 1995; Bosl and Motzer, 1997; Black et al, 1997; Kamdar et al, 1998). However, little information is available concerning geographic patterns of testicular cancer in smaller areas within a country. Scandinavian countries showed a marked homogeneity in testicular cancer incidence within each country, in contrast to the significant heterogeneity between the countries (Moller Jensen et al, 1988; Adami et al, 1994). In addition to nationwide geographic variations, differences in testicular cancer IRs have been observed between urban and rural communities. In Denmark, higher IRs of testicular cancer in men who grew up in urban areas have been reported (Clemmesen, 1968; Moller, 1997). On the other hand, an increased incidence of testicular cancer, in particular of seminomas, has been observed among men in rural communities in the UK and the US (Lipworth and Dayan, 1969; Graham et al, 1977; UK Testicular Cancer Study Group, 1994).

Within The Netherlands, a study in the 1960s showed a higher incidence of testicular neoplasms in a rural district of Friesland in the north compared with two urban communities in the western part of the country (Talerman et al, 1974). However, a more recent study, analysing urban–rural variations in cancer incidence within The Netherlands in the period 1989–1991, demonstrated no differences in testicular cancer IRs between urban and rural areas (Schouten et al, 1996).

In general, geographic variations and urban–rural differences in incidence of testicular cancer may be caused by differences in the inhabitants' personal behaviour and lifestyle patterns, exposure to environmental pollution and occupational hazards (Doll, 1991). Furthermore, geographic and urban–rural variations may also result from differences in genetic susceptibility to testicular cancer. The influence of genetic factors on cancer rates seems to

*Received 9 February 1999*
*Revised 7 June 1999*
*Accepted 7 June 1999*

*Correspondence to:* HJ Hoekstra

**Table 1** Total number of cases, age-adjusted IR per 100 000 men by histology, and IRR of testicular cancer in 12 provinces in The Netherlands in the period 1989–1995

| | | | Age-adjusted IR | | | | |
|---|---|---|---|---|---|---|---|
| **Survey area** | **Average male population** | **Total no. of cases** | **Sem** | **NS** | **Overall[a]** | **IRR[b]** | **95% CI** |
| Groningen | 274 404 | 125 | 3.1 | 2.4 | 5.8 | 1.3 | 1.1–1.6[c] |
| Friesland | 299 884 | 118 | 3.0 | 1.8 | 5.3 | 1.2 | 1.0–1.5 |
| Drenthe | 221 916 | 67 | 2.4 | 1.7 | 4.1 | 0.9 | 0.7–1.2 |
| Overijssel | 514 467 | 171 | 2.1 | 1.9 | 4.3 | 1.0 | 0.8–1.2 |
| Flevoland | 116 550 | 41 | 1.9 | 2.2 | 4.5 | 1.0 | 0.7–1.4 |
| Gelderland | 902 166 | 342 | 2.7 | 1.8 | 4.9 | 1.1 | 1.0–1.2 |
| Utrecht | 504 175 | 182 | 2.3 | 2.0 | 4.5 | 1.0 | 0.8–1.2 |
| Noord-Holland | 1 187 575 | 419 | 2.3 | 1.9 | 4.3 | 1.0 | 0.8–1.1 |
| Zuid-Holland | 1 607 425 | 537 | 2.3 | 1.7 | 4.2 | 1.0 | 0.8–1.1 |
| Zeeland | 178 319 | 45 | 1.8 | 1.5 | 3.4 | 0.8 | 0.5–1.1 |
| Noord-Brabant | 1 112 942 | 362 | 2.2 | 1.7 | 4.1 | 0.9 | 0.8–1.1 |
| Limburg | 553 853 | 179 | 2.1 | 1.9 | 4.2 | 1.0 | 0.8–1.1 |
| The Netherlands | 7 473 676 | 2591[d] | 2.3 | 1.8 | 4.4 | 1.0 | Reference |

Sem; seminoma, NS; non-seminoma. [a]Including cases with other histological type than seminoma or non-seminoma. [b]IR of overall histological types per province refered to IR in The Netherlands. [c]$P < 0.05$. [d]In three cases the residence was unknown.

be stronger in stable homogeneous populations, often living in rural areas, because members of these populations tend to be closely related and are likely to share genes from common ancestors, including possible disease-related genes (Talerman et al, 1974; Te Meerman et al, 1995).

In the present study, geographic patterns of testicular cancer IRs in the 12 provinces in The Netherlands during the period 1989–1995 are analysed. Moreover, the incidence of testicular cancer by degree of urbanization is evaluated. In addition, factors supporting a genetic susceptibility to testicular neoplasms and the suitability of stable founder populations for searching possible genes involved in the development of testicular cancer are discussed.

### PATIENTS AND METHODS

Data on age-adjusted incidence of testicular cancer in The Netherlands in the period 1989–1995 were retrieved from the files of the nationwide population-based Netherlands Cancer Registry (NCR). Since 1989, the NCR covers the whole population of The Netherlands. The main sources for the NCR are the computerized databanks of all pathology departments in the country (PALGA) and the hospital discharge databank to which all hospitals in the country yearly provide information on all discharge diagnoses of admitted patients. The overall completeness of the nationwide cancer databank of the NCR is estimated to be about 95% for all cancer sites (Schouten et al, 1993; Van der Sanden et al, 1995).

The Netherlands consist of a total of 12 provinces. In general, the majority of the Dutch population lives in urban municipalities in the western provinces of the country (Zuid-Holland, Noord-Holland, Utrecht), while particularly the northern provinces (Groningen, Friesland, Drenthe) are less densely populated.

Information about population figures was obtained from the Dutch Central Bureau of Statistics (CBS) (Central Bureau of Statistics, 1996). The average male population figures between 1989 and 1995 were based on population figures in 1990, 1992 and 1994. The CBS has also classified municipalities by level of urbanization according to an index based on the address density of the surroundings (Den Dulk et al, 1992).

Using this index, municipalities were classified into five groups with consecutive degrees of urbanization, from no urbanization to very dense urbanization. The total number of cases and age-adjusted IRs of testicular cancer per 100 000 men in each of 12 Dutch provinces in the period 1989–1995 were analysed. To provide information on the stability of the IRs, age-specific IRs of testicular cancer in six 15-year age groups were analysed. In addition, separate analyses were performed for the age-adjusted IRs of the two main histological types: seminoma and non-seminoma. For all histological types combined, incidence rate ratios (IRRs) were calculated by dividing the IR of testicular cancer in each province by the IR of testicular cancer in the whole country. IRs were age-adjusted by direct standardization to the European Standard Population. Bilateral testicular cancer cases attributed only one neoplasm to the calculation of IRs. The precision of IRRs was assessed by calculating 95% confidence intervals (CIs) using the method described by Rothman (1986). Increased regional IRs compared to the IR in The Netherlands were considered to be statistically significant whenever the lower limit of the 95% confidence interval (CI) did not include 1.00.

In addition, the age-adjusted IR of testicular cancer in relation to the degree of urbanization in The Netherlands during the period 1989–1995 was evaluated. Separate analyses were performed for the IRs of the two main histologic types, i.e. seminomas and non-seminomas, in the five urbanization classes.

### RESULTS

The average male population, the total number of cases and the age-adjusted IRs by histology in the 12 Dutch provinces in the period 1989–1995, as well as the IRR of the testicular cancer IR of each province refered to the IR of the whole country, are shown in Table 1. The 12 Dutch provinces are represented in Figure 1. The overall annual age-adjusted IR of testicular cancer in The Netherlands in the period 1989–1995 was 4.4 per 100 000 men. The annual age-adjusted IRs of the two main histological types, seminoma and non-seminoma, were 2.3 per 100 000 men and 1.8 per 100 000 men, respectively. The IR of other histological types (which did not include malignant lymphomas) was 0.2 per

*© 1999 Cancer Research Campaign* 

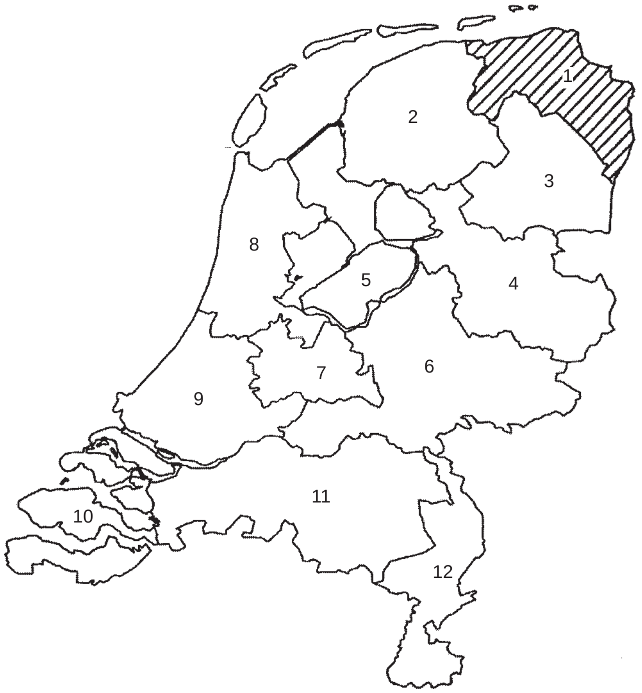

**Figure 1** Incidence rates (IR) of testicular cancer in 12 Dutch provinces referred to IR in The Netherlands. 1. Groningen; 2. Friesland; 3. Drenthe; 4. Overijssel; 5. Flevoland; 6. Gelderland; 7. Utrecht; 8. Noord-Holland; 9. Zuid-Holland; 10. Zeeland; 11. Noord-Brabant; 12. Limburg. (□) IR not different from IR in The Netherlands; (▨) IR significantly higher than IR in The Netherlands

**Table 2** Age-specific IR of testicular cancer per 100 000 men in 12 provinces in The Netherlands in the period 1989–1995

| | Age (years) | | | | | | |
|---|---|---|---|---|---|---|---|
| **Survey area** | **0–14** | **15–29** | **30–44** | **45–59** | **60–74** | **≥75** | **Overall** |
| Groningen | – | 9.3 | 11.8 | 5.5 | 3.2 | – | 5.8 |
| Friesland | 0.5 | 7.9 | 9.8 | 4.9 | 3.8 | 2.1 | 5.3 |
| Drenthe | 0.3 | 6.1 | 8.1 | 3.9 | 2.1 | – | 4.1 |
| Overijssel | 0.1 | 7.2 | 9.5 | 2.4 | 2.0 | 1.5 | 4.3 |
| Flevoland | – | 8.7 | 8.3 | 0.9 | 5.5 | 5.4 | 4.5 |
| Gelderland | 0.5 | 8.0 | 10.0 | 4.0 | 1.7 | 1.7 | 4.9 |
| Utrecht | 0.7 | 7.3 | 8.8 | 3.5 | 3.3 | 1.6 | 4.5 |
| Noord-Holland | 0.2 | 7.9 | 8.7 | 3.4 | 2.2 | 0.3 | 4.3 |
| Zuid-Holland | 0.3 | 7.6 | 8.9 | 3.1 | 0.7 | 1.5 | 4.2 |
| Zeeland | – | 6.8 | 6.4 | 2.7 | 1.3 | – | 3.4 |
| Noord-Brabant | 0.3 | 6.9 | 9.0 | 2.4 | 2.0 | 0.8 | 4.1 |
| Limburg | 0.1 | 8.1 | 7.4 | 3.3 | 1.7 | 1.6 | 4.2 |
| The Netherlands | 0.3 | 7.6 | 9.0 | 3.3 | 1.9 | 1.2 | 4.4 |

100 000 men. The province Groningen, in the northern part of the country, showed the highest IR of testicular cancer with 5.8 cases per 100 000 men per year. This IR was higher than the overall IR in The Netherlands (IRR 1.3, 95% CI 1.1–1.6, $P < 0.05$). In addition, Groningen showed the highest age-specific IRs in all relevant younger age-groups (15–29, 30–44 and 45–59 years; Table 2). Moreover, the highest IR in Groningen was seen for both seminomas (IR 3.1) and non-seminomas (IR 2.4). The high overall IR and the relative stability of this rate as it was based on high IRs in younger age-groups and for the main histological types indicate that the increased incidence in Groningen might be genuine. Friesland, also situated in the northern part of The Netherlands, showed the second highest IR of testicular cancer with 5.3 cases per 100 000 men per year (IRR 1.2, 95% CI 1.0–1.5, not significant). This mainly resulted from the high IR of seminoma in Friesland (IR 3.0). The lowest IR of testicular cancer was found in Zeeland, in the Southwest, with 3.4 cases per 100 000 men (IRR 0.8, 95% CI 0.5–1.1, not significant). The lowest IR in Zeeland was seen for both seminomas (IR 1.8) and non-seminomas (IR 1.5).

The avarage male population, the total number of cases and the age-adjusted IRs of testicular cancer by histology related to the degree of urbanization in The Netherlands are listed in Table 3. No differences were found between IRs in the five urbanization classes and the IR in The Netherlands at analyses of all histological types, or at separate analyses of seminomas and non-seminomas.

### DISCUSSION

Geographic clustering of testicular cancer within the northern part of a small country like The Netherlands is an interesting finding. In addition, no urban–rural differences in the incidence of testicular cancer were found in The Netherlands. Thus, although the north of The Netherlands predominantly consists of rural areas, the observed geographic clustering of testicular cancer in this part of the country most likely also results from other factors than those directly related to the degree of urbanization. In general, clues to

**Table 3** Total number of cases with testicular cancer and age-adjusted IR per 100 000 men by histology related to the degree of urbanization in The Netherlands in the period 1989–1995

| | | | | Age-adjusted IR | | |
|---|---|---|---|---|---|---|
| **Degree of urbanization (No. of addresses km$^{-2}$)** | **Average male population** | **Population density (n km$^{-2}$)** | **Total no. of cases (%)** | **Sem** | **NS** | **Overall[a]** |
| Non-urban (<500) | 1 453 093 | 161 | 476 (18) | 2.3 | 1.8 | 4.4 |
| Sparse (500–<1000) | 1 547 943 | 338 | 561 (22) | 2.5 | 2.0 | 4.7 |
| Moderate (1000–<1500) | 1 545 788 | 798 | 578 (22) | 2.6 | 2.1 | 4.9 |
| Dense (1500–<2500) | 1 558 140 | 1 731 | 505 (20) | 2.1 | 1.7 | 4.1 |
| Very dense (≤2500) | 1 368 712 | 3 791 | 468 (18) | 2.1 | 1.6 | 3.8 |
| Total | 7 473 676 | 449 | 2591[b] | 2.3 | 1.8 | 4.4 |

Sem; seminoma, NS; non-seminoma. [a]Including cases with other histologic type than seminoma or non-seminoma. [b]In three cases the residence was unknown.

 *© 1999 Cancer Research Campaign*

causes of cancer can often be generated from its geographic patterns of occurrence (Blot and Fraumeni, 1978). It is difficult, however, to make definite inferences from single geographic clustering of neoplasms if aetiological factors of the neoplasm are largely unknown. This is of particular concern for testicular cancer, for which aetiology is poorly understood. A history of undescended testis is the most established risk factor. Other risk factors include a family history of testicular cancer, in utero exposure to oestrogens, infertility, a low birth weight and conditions of an abnormal sexual differentiation (Savage and Lowe, 1990; Sharpe and Skakkebaek, 1993; Akre et al, 1996; Dieckmann and Pichlmeier, 1997; Moller and Skakkebaek, 1999). In view of the poorly understood aetiology, it is difficult to speculate about the aetiological significance of the regional clustering of testicular cancer as found in the present series.

In general, clustering of cancer within a population may result from shared environment, shared genes, or both. Theoretically, it is also possible that geographic clustering of cancer results from a combination of random factors. Furthermore, bias due to incomplete case ascertainment, errors in case registration and inaccurate residential information could cause geographic disease clustering. Moreover, bias may occur due to regional differences in patient presentation, although this seems less likely for testicular cancer than for other cancers, regarding the peak of testicular cancer in young age. It must also be noted that the geographic clustering represents a random finding in the population in time. In the present series no information is available on place of birth, degree of migration and mobility of the population. Theoretically, this information might be of particular importance in testicular cancer as the population affected is relatively young and, consequently, is likely to move more often than the general population. In view of the lack of information on migration and mobility, the present findings have to be interpreted cautiously. On the other hand, however, the high incidence of testicular cancer in the province Groningen in the north was demonstrated for both seminomas and non-seminomas, and was additionally found in all relevant young age groups in which testicular cancer most frequently occurs. These consistent findings indicate that the high incidence figure of testicular cancer in the north is rather stable.

A wide range of environmental factors has been associated with an increased risk to develop testicular cancer (Mills et al, 1984; UK Testicular Cancer Study Group, 1994; Kristensen et al, 1996; Hardell et al, 1997; Moller, 1997). However, many of the associations show little consistency and, consequently, have to be judged with some reserve. Moreover, none of the associations between environmental factors and testicular cancer development has been convincingly correlated with geographic variations in the incidence of testicular cancer (Adami et al, 1994).

In addition to possible common environmental factors, geographic clustering of testicular cancer may also result from a common genetic susceptibility to the disease. This seems of particular importance in stable founder populations which tend to be closely related and are likely to share possible disease-related genes from common ancestors (Talerman et al, 1974; Te Meerman et al, 1995). Several other observations point to a role of genetic factors in the aetiology of testicular cancer. Familial and bilateral testicular cancer cases occur more frequently than expected by chance. Familial aggregation of testicular neoplasms is reported in 1.0–2.8% of cases. Moreover, the relative risk to brothers of testicular cancer cases is increased by a factor of 3–13 (Dieckmann and Pichlmeier, 1997; Sonneveld et al, 1999). In the absence of potent environmental risk factors for testicular cancer, this indicates the involvement of genetic factors in the aetiology of the disease (Khoury et al, 1988). The prevalence of bilateral testicular cancer in patients with unilateral testicular tumours varies between 1.0% and 5.8% (Osterlind et al, 1991; Colls et al, 1996; Sonneveld et al, 1998). Patients with bilateral involvement of paired organs, including the testes, are generally considered to be at high risk of having a genetic predisposition to the disease. A genetic susceptibility is emphasized by an increased incidence of testicular cancer in individuals with certain rare malformations of the urogenital system, some of which have a definite genetic component in the aetiology (Savage and Lowe, 1990; Heimdal and Fossa, 1994). Moreover, higher rates of urogenital developmental anomalies have been reported in families prone to testicular cancer (Tollerud et al, 1985; Sonneveld et al, 1999). Furthermore, testicular neoplasms are usually diagnosed at a young age, i.e. in early adult life, and the incidence declines after the age of 50 years. The young age at onset of testicular cancer indicates a role of important aetiologic factors operating early in life, such as in utero exposure to maternal oestrogens or exposure to infectious agents in early childhood (Mills et al, 1984; Newell et al, 1984; Heimdal and Fossa, 1994). Early operating aetiologic factors, however, may also include genetic influences. In addition to the literature, the geographic clustering of testicular neoplasms in the present series may also lend support to a genetic susceptibility. Demonstration that a disease occurs more frequently among individuals related by common ancestry is a strong indication that genetic factors are involved in the aetiology of the disease (Hauck and Martin, 1984; Muntoni et al, 1997). Hypothetically, a genetic susceptibility to testicular cancer development seems likely among patients in the stable founder populations in the northern provinces of The Netherlands, showing higher incidence rates of testicular cancer than the mixed populations in the more urban areas of the country. The populations in the northern part of The Netherlands are formed relatively recently, i.e. about 2000 years ago, and mainly originate from the present day's Central European countries and Germany (Cavalli-Sforza et al, 1994). They have been stable over the past two millenia due to low mobility of people and low immigration. Consequently, these stable founder populations in the rural north are likely to have a relatively higher frequency of genes identical by descent than the mixed populations in most urban areas. Since these shared genes among descendants may also include possible genes predisposing to testicular neoplasms, the magnitude of a genetic susceptibility to testicular cancer is probably stronger in such homogeneous founder populations than in urban populations which tend to have a higher degree of genetic heterogeneity. The regional clustering of testicular cancer in the north of The Netherlands therefore may lend support to the involvement of genetic factors in testicular cancer development, at least in part of the patients.

An observation by others which may be of interest in view of the present findings, is the small genetic distance existing between the Dutch and Danish population, indicating a similar origin of both populations (Cavalli-Sforza et al, 1994). The incidence of testicular cancer in Denmark is about twice as high as in The Netherlands (Adami et al, 1994; Buetow, 1995). However, since the north of The Netherlands is geographically closest to Denmark, the genetic homogeneity between the Dutch and Danish population is likely to be strongest for the Dutch population in the north. Regarding the relatively high testicular cancer IR in this part of The Netherlands, hypothetically this might indicate a possible common genetic susceptibility.

*© 1999 Cancer Research Campaign*

Thus, based on findings in numerous clinical and epidemiologic studies, including the current study, there is sufficient evidence to postulate a genetic susceptibility to testicular cancer. Although several candidate genes have been proposed, further research is indicated to clarify the molecular genetic basis and to identify the testicular cancer susceptibility gene(s) (Leahy et al, 1995; International Testicular Cancer Linkage Consortium, 1998; Murty and Chaganti, 1998). Individuals in stable founder populations share a relatively high frequency of genes from common ancestors, i.e. genes which are identical by descent (Te Meerman et al, 1995). Genes possibly predisposing to testicular cancer are likely to show increased frequencies among affected men in these founder populations. Consequently, as testicular cancer is rare and mapping of disease genes is difficult, testicular cancer cases in founder populations seem to be more suitable for searching genes involved in the development of testicular cancer than cases from general populations.

In conclusion, geographic clustering of testicular cancer seems to be present in the northern part of The Netherlands in areas with some stable founder populations. This finding may lend support to a genetic susceptibility to testicular cancer development, although it does not exclude the involvement of shared environmental factors in the aetiology of the disease. Therefore, in addition to studying the role of aetiologic environmental factors, further research is indicated to identify potential susceptibility genes. Testicular cancer cases in stable founder populations seem particularly suitable for searching genes predisposing to the development of testicular cancer.

### ACKNOWLEDGEMENTS

This research was supported by a preclinical fellowship of the Dutch Cancer Society.

### REFERENCES

Adami H-O, Bergstrom R, Mohner M, Zatonski W, Storm H, Ekbom A, Tretli S, Teppo L, Ziegler H, Rahu M, Gurevicius R and Stengrevics A (1994) Testicular cancer in nine northern European countries. *Int J Cancer* **59**: 33–38

Akre O, Ekbom A, Hsieh CC, Trichopoulos D and Adami H-O (1996) Testicular non-seminoma and seminoma in relation to perinatal characteristics. *J Natl Cancer Inst* **88**: 883–889

Black RJ, Bray F, Ferlay J and Parkin DM (1997) Cancer incidence and mortality in the European Union: cancer registry data and estimates of national incidence for 1990. *Eur J Cancer* **33**: 1075–1107

Blot WJ and Fraumeni JF (1979) Geographic patterns of renal cancer in the United States. *J Natl Cancer Inst* **63**: 363–366

Bosl GJ and Motzer RJ (1997) Testicular germ cell cancer. *New Engl J Med* **337**: 242–253

Buetow SA (1995) Epidemiology of testicular cancer. *Epidem Rev* **17**: 433–449

Cavalli-Sforza LL, Menozzi P and Piazza A (1994) Europe. In: *The History and Geography of Human Genes,* Cavalli-Sforza LL, Menozzi P and Piazza A (eds), p 255–301. Princeton University Press: Princeton

Central Bureau of Statistics (1996) *Population of Dutch Municipalities 1989–1995.* SDU/CBS: Gravenhage

Clemmesen J (1968) A doubling of morbidity from testis carcinoma in Copenhagen, 1943–1962. *Acta Path Microbiol Scand* **72**: 348–349

Colls BM, Harvey VJ, Skelton L, Thompson PI and Frampton CM (1996) Bilateral germ cell tumours in New Zealand: experience in Auckland and Christchurch 1978–1994. *J Clin Oncol* **14**: 2061–2065

Den Dulk CJ, Van der Stadt H and Vliegen JM (1992) A new measure for degree of urbanisation: the address density of the surrounding area. *Mndstat Bevolk (CBS)* **7**: 14–27

Dieckmann KP and Pichlmeier U (1997) The prevalence of familial testicular cancer. An analysis of two patient populations and a review of the literature. *Cancer* **80**: 1954–1960

Doll R (1991) Urban and rural factors in the aetiology of cancer. *Int J Cancer* **47**: 803–810

Graham S, Gibson R, West D, Swanson M, Burnett W and Dayal H (1977) Epidemiology of cancer of the testis in upstate New York. *J Natl Cancer Inst* **58**: 1255–1261

Hardell L, Ohlson CG and Fredrikson M (1997) Occupational exposure to polyvinyl chloride as a risk factor for testicular cancer evaluated in a case–control study. *Int J Cancer* **73**: 828–830

Hauck WW and Martin AO (1984) A statistical test for detection of ancestral genetic contributions to disease occurrence in finite populations. *Gen Epidemiol* **1**: 383–400

Heimdal K and Fossa SD (1994) Genetic factors in malignant germ-cell tumours. *World J Urol* **12**: 178–181

International Testicular Cancer Linkage Consortium (1998) Candidate regions for testicular cancer susceptibility genes. *APMIS* **106**: 64–72

Kamdar RH, Oliver RTD, Othieno-Abinya N, Gallagher CJ and Slevin ML (1998) Geographical epidemiology of ovarian and testicular germ cell cancers. *Br J Cancer* **78**: 1401

Khoury MJ, Beaty TH and Liang K (1988) Can familial aggregation of disease be explained by familial aggregation of environmental risk factors? *Am J Epidemiol* **127**: 674–683

Kolonel LN, Ross RK, Thomas DB and Thompson DJ (1982) Epidemiology of testicular cancer in the Pacific basin. *Natl Cancer Inst Monogr* **62**: 157–160

Kristensen P, Andersen A, Irgens LM, Bye AS and Vagstad N (1996) Testicular cancer and parental use of fertilizers in agriculture. *Cancer Epidemiol Biomarkers Prev* **5**: 3–9

Leahy MG, Tonks S, Moses JH, Brett AR, Huddart R, Forman D, Oliver RTD, Bishop DT and Bodmer JG (1995) Candidate regions for a testicular cancer susceptibility gene. *Hum Mol Genet* **4**: 1551–1555

Lipworth L and Dayan AD (1969) Rural preponderance of seminoma of the testis. *Cancer* **23**: 1119–1121

Mills PK, Newell GR and Johnson DE (1984) Testicular cancer associated with employment in agriculture and oil and natural gas extraction. *Lancet* **i**: 207–210

Moller H (1997) Work in agriculture, childhood residence, nitrate exposure, and testicular cancer risk: a case–control study in Denmark. *Cancer Epidemiol Biomarkers Prev* **6**: 141–144

Moller H and Skakkebaek NE (1999) Risk of testicular cancer in subfertile men: case–control study. *BMJ* **318**: 559–562

Moller Jensen O, Carstensen B, Glattre E, Malker B, Pukkala E and Tulinius H (1988) *Atlas of cancer incidence in Nordic countries*. Nordic Cancer Union: Helsinki

Muntoni SA, Fonte MT, Stoduto S, Marietti G, Bizzarri C, Crino A, Ciampalini P, Multari G, Suppa MA, Matteoli MC, Lucentini L, Sebatiani LM, Visalli N, Pozzilli P, Boscherini B and Muntoni S (1997) Incidence of insulin-dependent diabetes mellitus among Sardinian-heritage children born in Lazio region, Italy. *Lancet* **349**: 160–162

Murty VVVS and Chaganti RSK (1998) A genetic perspective of male germ cell tumours. *Semin Oncol* **25**: 133–144

Newell GR, Mills PK and Johnson DE (1984) Epidemiologic comparison of cancer of the testis and Hodgkin's disease among young males. *Cancer* **54**: 1117–1123

Osterlind A, Berthelsen JG, Abildgaard N, Hansen SO, Hjalgrim H, Johansen B, Munck-Hansen J and Rasmussen LH (1991) Risk of bilateral germ cell cancer in Denmark: 1960–1984. *J Natl Cancer Inst* **83**: 1391–1395

Rothman KJ (1986) *Modern Epidemiology*, p 117. Little, Brown: Boston

Savage MO and Lowe DG (1990) Gonadal neoplasia and abnormal sexual differentiation. *Clin Endocrin* **32**: 519–533

Schouten LJ, Hoppener P, Van den Brandt PA, Knottnerus JA and Jager JJ (1993) Completeness of cancer registration in Limburg. The Netherlands. *Int J Epidemiol* **22**: 369–376

Schouten LJ, Meijer H, Huveneers JAM and Kiemeney LALM (1996) Urban–rural differences in cancer incidence in The Netherlands, 1989–1991. *Int J Epidemiol* **25**: 729–736

Sharpe RM and Skakkebaek NE (1993) Are oestrogens involved in falling sperm counts and disorders of the male reproductive tract? *Lancet* **341**: 1392–1395

Sonneveld DJA, Sleijfer DTh, Schraffordt Koops H, Sijmons RH, Van der Graaf WTA, Sluiter WJ and Hoekstra HJ (1999) Familial testicular cancer in a single centre population. *Eur J Cancer (in press)*

Sonneveld DJA, Schraffordt Koops H, Sleijfer DTh and Hoekstra HJ (1998) Bilateral testicular germ cell tumours in patients with initial stage I disease: prevalence and prognosis – a single centre's 30 years' experience. *Eur J Cancer* **34**: 1363–1367

Talerman A, Kaalen JGAH and Fokkens W (1974) Rural preponderance of testicular

 *© 1999 Cancer Research Campaign*

neoplasms. *Br J Cancer* **29**: 176–178

Te Meerman GJ, Van der Meulen MA and Sandkuijl LA (1995) Perspectives of identity by descent (IBD) mapping in founder populations. *Clin Exp Allergy* **25**: 97–102

Tollerud DJ, Blattner WA, Fraser MC, Morris Brown L, Pottern LM, Shapiro E, Kirkemo A, Shawker TH, Javadpour N, O'Connell K, Stutzman RE and Fraumeni EF (1985) Familial testicular cancer and urogenital developmental anomalies. *Cancer* **55**: 1849–1854

Van der Sanden GAC, Coebergh JWW, Schouten LJ, Visser O and Van Leeuwen FE (1995) Cancer incidence in The Netherlands in 1989 and 1990: first results of the nationwide Netherlands Cancer Registry. *Eur J Cancer* **31A**: 1822–1829

UK Testicular Cancer Study Group (1994) Social, behavioural and medical factors in the aetiology of testicular cancer: results from the UK study. *Br J Cancer* **70**: 513–520

Wilkinson TJ, Colls BM and Schluter PJ (1992) Increased incidence of germ cell testicular cancer in New Zealand Maoris. *Br J Cancer* **65**: 769–771

*© 1999 Cancer Research Campaign*

## References

[PDF_00434] Adami H. O., Bergström R., Möhner M., Zatoński W., Storm H., Ekbom A., Tretli S., Teppo L., Ziegler H., Rahu M. (1994). Testicular cancer in nine northern European countries.. Int J Cancer.

[PDF_00437] Akre O., Ekbom A., Hsieh C. C., Trichopoulos D., Adami H. O. (1996). Testicular nonseminoma and seminoma in relation to perinatal characteristics.. J Natl Cancer Inst.

[PDF_00440] Black R. J., Bray F., Ferlay J., Parkin D. M. (1997). Cancer incidence and mortality in the European Union: cancer registry data and estimates of national incidence for 1990.. Eur J Cancer.

[PDF_00443] Blot W. J., Fraumeni J. F. (1979). Geographic patterns of renal cancer in the United States.. J Natl Cancer Inst.

[PDF_00445] Bosl G. J., Motzer R. J. (1997). Testicular germ-cell cancer.. N Engl J Med.

[PDF_00447] Buetow S. A. (1995). Epidemiology of testicular cancer.. Epidemiol Rev.

[PDF_00453] Clemmesen J. (1968). A doubling of morbidity from testis carcinoma in Copenhagen, 1943-1962.. Acta Pathol Microbiol Scand.

[PDF_00455] Colls B. M., Harvey V. J., Skelton L., Thompson P. I., Frampton C. M. (1996). Bilateral germ cell testicular tumors in New Zealand: experience in Auckland and Christchurch 1978-1994.. J Clin Oncol.

[PDF_00458] Den Dulk C. J., Van De Stadt H., Vliegen J. M. (1992). Een nieuwe maatstaf voor stedelijkheid: de omgevingsadressendichtheid.. Maandstat Bevolking.

[PDF_00461] Dieckmann K. P., Pichlmeier U. (1997). The prevalence of familial testicular cancer: an analysis of two patient populations and a review of the literature.. Cancer.

[PDF_00464] Doll R. (1991). Urban and rural factors in the aetiology of cancer.. Int J Cancer.

[PDF_00466] Graham S., Gibson R., West D., Swanson M., Burnett W., Dayal H. (1977). Epidemiology of cancer of the testis in upstate New York.. J Natl Cancer Inst.

[PDF_00469] Hardell L., Ohlson C. G., Fredrikson M. (1997). Occupational exposure to polyvinyl chloride as a risk factor for testicular cancer evaluated in a case-control study.. Int J Cancer.

[PDF_00472] Hauck W. W., Martin A. O. (1984). A statistical test for detection of ancestral genetic contributions to disease occurrence in finite populations.. Genet Epidemiol.

[PDF_00475] Heimdal K., Fosså S. D. (1994). Genetic factors in malignant germ-cell tumors.. World J Urol.

[PDF_00479] Kamdar R. H., Oliver R. T., Othieno-Abinya N., Gallagher C. J., Slevin M. L. (1998). Geographical epidemiology of ovarian and testicular germ cell cancers.. Br J Cancer.

[PDF_00482] Khoury M. J., Beaty T. H., Liang K. Y. (1988). Can familial aggregation of disease be explained by familial aggregation of environmental risk factors?. Am J Epidemiol.

[PDF_00485] Kolonel L. N., Ross R. K., Thomas D. B., Thompson D. J. (1982). Epidemiology of testicular cancer in the Pacific Basin.. Natl Cancer Inst Monogr.

[PDF_00487] Kristensen P., Andersen A., Irgens L. M., Bye A. S., Vagstad N. (1996). Testicular cancer and parental use of fertilizers in agriculture.. Cancer Epidemiol Biomarkers Prev.

[PDF_00490] Leahy M. G., Tonks S., Moses J. H., Brett A. R., Huddart R., Forman D., Oliver R. T., Bishop D. T., Bodmer J. G. (1995). Candidate regions for a testicular cancer susceptibility gene.. Hum Mol Genet.

[PDF_00493] Lipworth L., Dayan A. D. (1969). Rural preponderance of seminoma of the testis.. Cancer.

[PDF_00495] Mills P. K., Newell G. R., Johnson D. E. (1984). Testicular cancer associated with employment in agriculture and oil and natural gas extraction.. Lancet.

[PDF_00505] Muntoni S., Fonte M. T., Stoduto S., Marietti G., Bizzarri C., Crinò A., Ciampalini P., Multari G., Suppa M. A., Matteoli M. C. (1997). Incidence of insulin-dependent diabetes mellitus among Sardinian-heritage children born in Lazio region, Italy.. Lancet.

[PDF_00511] Murty V. V., Chaganti R. S. (1998). A genetic perspective of male germ cell tumors.. Semin Oncol.

[PDF_00501] Møller H., Skakkebaek N. E. (1999). Risk of testicular cancer in subfertile men: case-control study.. BMJ.

[PDF_00498] Møller H. (1997). Work in agriculture, childhood residence, nitrate exposure, and testicular cancer risk: a case-control study in Denmark.. Cancer Epidemiol Biomarkers Prev.

[PDF_00513] Newell G. R., Mills P. K., Johnson D. E. (1984). Epidemiologic comparison of cancer of the testis and Hodgkin's disease among young males.. Cancer.

[PDF_00516] Osterlind A., Berthelsen J. G., Abildgaard N., Hansen S. O., Hjalgrim H., Johansen B., Munck-Hansen J., Rasmussen L. H. (1991). Risk of bilateral testicular germ cell cancer in Denmark: 1960-1984.. J Natl Cancer Inst.

[PDF_00520] Savage M. O., Lowe D. G. (1990). Gonadal neoplasia and abnormal sexual differentiation.. Clin Endocrinol (Oxf).

[PDF_00522] Schouten L. J., Höppener P., van den Brandt P. A., Knottnerus J. A., Jager J. J. (1993). Completeness of cancer registration in Limburg, The Netherlands.. Int J Epidemiol.

[PDF_00525] Schouten L. J., Meijer H., Huveneers J. A., Kiemeney L. A. (1996). Urban-rural differences in cancer incidence in The Netherlands 1989-1991.. Int J Epidemiol.

[PDF_00528] Sharpe R. M., Skakkebaek N. E. (1993). Are oestrogens involved in falling sperm counts and disorders of the male reproductive tract?. Lancet.

[PDF_00530] Sonneveld D. J., Schraffordt Koops H., Sleijfer D. T., Hoekstra H. J. (1998). Bilateral testicular germ cell tumours in patients with initial stage I disease: prevalence and prognosis--a single centre's 30 years' experience.. Eur J Cancer.

[PDF_00537] Talerman A., Kaalen J. G., Fokkens W. (1974). Rural preponderance of testicular neoplasms.. Br J Cancer.

[PDF_00543] Te Meerman G. J., Van der Meulen M. A., Sandkuijl L. A. (1995). Perspectives of identity by descent (IBD) mapping in founder populations.. Clin Exp Allergy.

[PDF_00546] Tollerud D. J., Blattner W. A., Fraser M. C., Brown L. M., Pottern L., Shapiro E., Kirkemo A., Shawker T. H., Javadpour N., O'Connell K. (1985). Familial testicular cancer and urogenital developmental anomalies.. Cancer.

[PDF_00556] Wilkinson T. J., Colls B. M., Schluter P. J. (1992). Increased incidence of germ cell testicular cancer in New Zealand Maoris.. Br J Cancer.

[PDF_00550] van der Sanden G. A., Coebergh J. W., Schouten L. J., Visser O., van Leeuwen F. E. (1995). Cancer incidence in The Netherlands in 1989 and 1990: first results of the nationwide Netherlands cancer registry. Coordinating Committee for Regional Cancer Registries.. Eur J Cancer.

